# Role of radiomic analysis of [^18^F]fluoromethylcholine PET/CT in predicting biochemical recurrence in a cohort of intermediate and high risk prostate cancer patients at initial staging

**DOI:** 10.1007/s00330-023-09642-9

**Published:** 2023-04-20

**Authors:** Francesca Marturano, Priscilla Guglielmo, Andrea Bettinelli, Fabio Zattoni, Giacomo Novara, Alessandra Zorz, Matteo Sepulcri, Michele Gregianin, Marta Paiusco, Laura Evangelista

**Affiliations:** 1 Department of Medical Physics, Veneto Institute of Oncology IOV - IRCCS, Padua, Italy; 2 Nuclear Medicine Unit, Veneto Institute of Oncology IOV - IRCCS, Padua, Italy; 3 Department of Information Engineering, University of Padua, Padua, Italy; 4 Department of Surgical Oncological & Gastroenterological Sciences (DiSCOG), University of Padua, Padua, Italy; 5 Department of Surgery, Oncology and Gastroenterology, University of Padua, Padua, Italy; 6 Radiotherapy Unit, Veneto Institute of Oncology IOV - IRCCS, Padua, Italy; 7 Nuclear Medicine Unit, Department of Medicine DIMED, University of Padua, Padua, Italy

**Keywords:** Prostatic neoplasms, Artificial intelligence, Fluorocholine, Positron emission tomography computed tomography

## Abstract

**Aim:**

To study the feasibility of radiomic analysis of baseline [^18^F]fluoromethylcholine positron emission tomography/computed tomography (PET/CT) for the prediction of biochemical recurrence (BCR) in a cohort of intermediate and high-risk prostate cancer (PCa) patients.

**Material and methods:**

Seventy-four patients were prospectively collected. We analyzed three prostate gland (PG) segmentations (i.e., PG_whole_: whole PG; PG_41%_: prostate having standardized uptake value – SUV > 0.41*SUVmax; PG_2.5_: prostate having SUV > 2.5) together with three SUV discretization steps (i.e., 0.2, 0.4, and 0.6). For each segmentation/discretization step, we trained a logistic regression model to predict BCR using radiomic and/or clinical features.

**Results:**

The median baseline prostate-specific antigen was 11 ng/mL, the Gleason score was > 7 for 54% of patients, and the clinical stage was T1/T2 for 89% and T3 for 9% of patients. The baseline clinical model achieved an area under the receiver operating characteristic curve (AUC) of 0.73. Performances improved when clinical data were combined with radiomic features, in particular for PG_2.5_ and 0.4 discretization, for which the median test AUC was 0.78.

**Conclusion:**

Radiomics reinforces clinical parameters in predicting BCR in intermediate and high-risk PCa patients. These first data strongly encourage further investigations on the use of radiomic analysis to identify patients at risk of BCR.

**Clinical relevance statement:**

The application of AI combined with radiomic analysis of [^18^F]fluoromethylcholine PET/CT images has proven to be a promising tool to stratify patients with intermediate or high-risk PCa in order to predict biochemical recurrence and tailor the best treatment options.

**Key Points:**

• *Stratification of patients with intermediate and high-risk prostate cancer at risk of biochemical recurrence before initial treatment would help determine the optimal curative strategy.*

• *Artificial intelligence combined with radiomic analysis of [*^*18*^*F]fluorocholine PET/CT images allows prediction of biochemical recurrence, especially when radiomic features are complemented with patients’ clinical information (highest median AUC of 0.78).*

• *Radiomics reinforces the information of conventional clinical parameters (i.e., Gleason score and initial prostate-specific antigen level) in predicting biochemical recurrence.*

**Supplementary Information:**

The online version contains supplementary material available at 10.1007/s00330-023-09642-9.

## Introduction

Prostate cancer (PCa) is the most frequently diagnosed cancer in men and the fifth leading cause of death worldwide [1, 2], even though its mortality rates have decreased in most high-income countries since the mid-1990s, thanks to improvement in earlier stage detection and therapeutic options [2].

In PCa, risk stratification at staging is crucial to determine the optimal treatment strategies and, therefore, prognosis. The 5-year risk stratification in patients with primary PCa is mainly based on clinical stage, baseline prostate-specific antigen (PSA) level, and Gleason score (GS) [3]. However, biopsy sampling is prone to incorrectly grade PCa, often resulting in undergrading [4], and minor complications, such as gross hematuria, hematospermia, and rectal bleeding, may occur [5, 6]. The recent introduction of magnetic resonance imaging (MRI) fusion-guided biopsy has significantly improved the detection of primary tumors, although the agreement between MRI and biopsy is sub-optimal and the entire whole gland cannot still be assessed before the radical prostatectomy (RP). Although primary treatments, either RP or curative radiotherapy (RT), 2–50% of patients experience a biochemical recurrence (BCR) within 10 years from therapy [7–9], defined as an increase in the serum PSA level above 0.2 ng/mL after RP, or a serum PSA level > nadir + 2.0 ng/mL after definitive radiotherapy [10]. Therefore, the assessment of BCR’s risk before initial treatment would be essential to planning the appropriate treatment approach.

Positron emission tomography (PET) combined with computed tomography (CT) or MRI using several radiotracers targeting choline (e.g., [^18^F]fluoromethylcholine and [^11^C]choline), prostate-specific membrane antigen-PSMA (labeled with ^68^Ga or ^18^F), and [^18^F]Fluciclovine can help to localize suspicious lesions in the prostate gland (PG), providing a valuable tool for the detection of cancer and thus to guide biopsies and treatment. Since its introduction, PET has been proved to be a fundamental examination at the initial staging of disease [11, 12] and it is currently recommended by several guidelines, especially in case of high-risk PCa [3, 13, 14]. A major advantage of imaging relies on the possibility to non-invasively and repeatedly sample an entire volume (whole tumor and/or any metastases), revealing its phenotypic characteristics over time, thus overcoming the invasiveness and sampling errors of biopsy [15].

In this context, artificial intelligence (AI) offers a promising adjunct to assist physicians in the analysis and interpretation of biomedical images, by performing tasks that would typically require human intelligence [16]. In oncology, AI-based models are often fed with features extracted from biomedical images, e.g., the radiomic features, combined with other clinical, demographic, and/or histopathological parameters, to build predictive or prognostic mathematical models of clinical outcomes, such as overall survival, recurrence, risk factor, and others. Specifically, radiomics is an evolving field in which large amounts of quantitative features are extracted from diagnostic medical images. These features may provide information linked to the underlying molecular and genetic characteristics, and thereby could be used to improve treatment response prediction and prognostication and potentially to allow personalisation of cancer treatment [15]. In particular, there is increasing interest in extracting additional characteristics from PET images that describe the heterogeneity of voxel intensities, that might be only subjectively measured or even missed by an expert eye, thereby providing additional, potentially relevant diagnostic information for clinical decision-making in a non-invasive manner [17, 18].

A recent review of PET radiomics shows that, although some published studies have limited robustness and reproducibility because of small amount of data available (< 50 of patients for the 30%) and miss validation on external datasets or in an independent subsample of the initial dataset (for 28%), the interest in PET radiomics is increasing exponentially [19]. The majority of these studies have concentrated mostly on lung, head, and neck, and gynecological cancers, likely as a consequence of their diffusion, while data about PCa and PET radiomics are still limited [19, 20]. To the best of our knowledge, in PCa patients, radiomic analysis has been investigated at initial staging, for recurrence detection, and in the case of metastatic disease by using mainly MRI [21]. However, PET radiomics has been shown to hold great potential in the assessment of tumor characterization, diagnosis, and prognosis [19].

The aim of the present study is to perform a radiomic analysis of [^18^F]fluoromethylcholine PET/CT images in a cohort of intermediate and high-risk PCa patients, in order to predict BCR. Since radiomics is demanding in terms of data and large cohorts of subjects are not always available, we implemented a robust internal validation pipeline.

## Materials and methods

Patients considered in the present study are part of a prospective trial (EUDRAcT number: 2013–002,511-99). All patients gave their informed consent for the use of their personal and clinical data. All procedures performed were in accordance with the ethical standards defined by the 1964 Helsinki Declaration and its later amendments.

### Patient population

For the study, we prospectively selected patients with an intermediate and high-risk PCa (according to the National Comprehensive Cancer Network-NCCN classification [22]) who underwent [^18^F]fluoromethylcholine PET/CT for initial staging of disease at the Veneto Institute of Oncology (Padua, Italy) from March 2013 to October 2019. The following inclusion criteria were used: (1) a confirmed intermediate- to high-risk PCa defined accordingly to the most recent EAU (European Association of Urologists) risk group classification (PSA levels greater than 10 ng/mL or Gleason Score > 7(4 + 3) or at least a cT2b clinical stage) [22, 23]; (2) age > 18 years; (3) patients who were candidates for radical prostatectomy and lymphadenectomy or radical radiotherapy; (4) accessible follow-up information; and (5) no visible CT artifacts due to implants. Conversely, patients with a previous history of cancer and/or patients who were pre-treated with hormone therapy were excluded. The final database included 74 consecutive patients (median age: 73 years, range [43–86]). Baseline clinical, demographic, and biological data, such as age, PSA, histological subtype, pre-surgery GS, clinical stage, and BCR were retrieved from medical records. Of these, baseline PSA, pre-surgery GS, and clinical stage were considered in the analysis. Missing clinical values were imputed with the k-Nearest Neighbors algorithm. To be included in the model, GS, and clinical stage were dichotomized: GS ≤ 7 versus GS > 7 and T1/T2 versus T3, respectively.

### PET/CT acquisition, reconstruction, and interpretation

A whole-body PET/CT was acquired from the skull vertex to the proximal femur, with 6–7 beds, 2–3 min per bed, 60 min after intravenous administration of the tracer (3 MBq/kg of [^18^F]fluoromethylcholine). A low-dose whole-body CT scan (with no contrast enhancement; 140 kV, 80–120 mA) was used for attenuation correction and for the anatomical localization of the sites of disease. The PET data were reconstructed with an in-plane voxel size of 4 mm and a slice thickness of 2 or 4 mm. The processed images were displayed in coronal, transverse, and sagittal planes. [^18^F]Fluoromethylcholine PET/CT images were jointly interpreted by two specialists trained to perform PET/CT imaging. The primary tumor was assessed by analyzing the whole PG and identifying areas with a focal tracer uptake.

### Prostate delineations

**Fig. 1:**
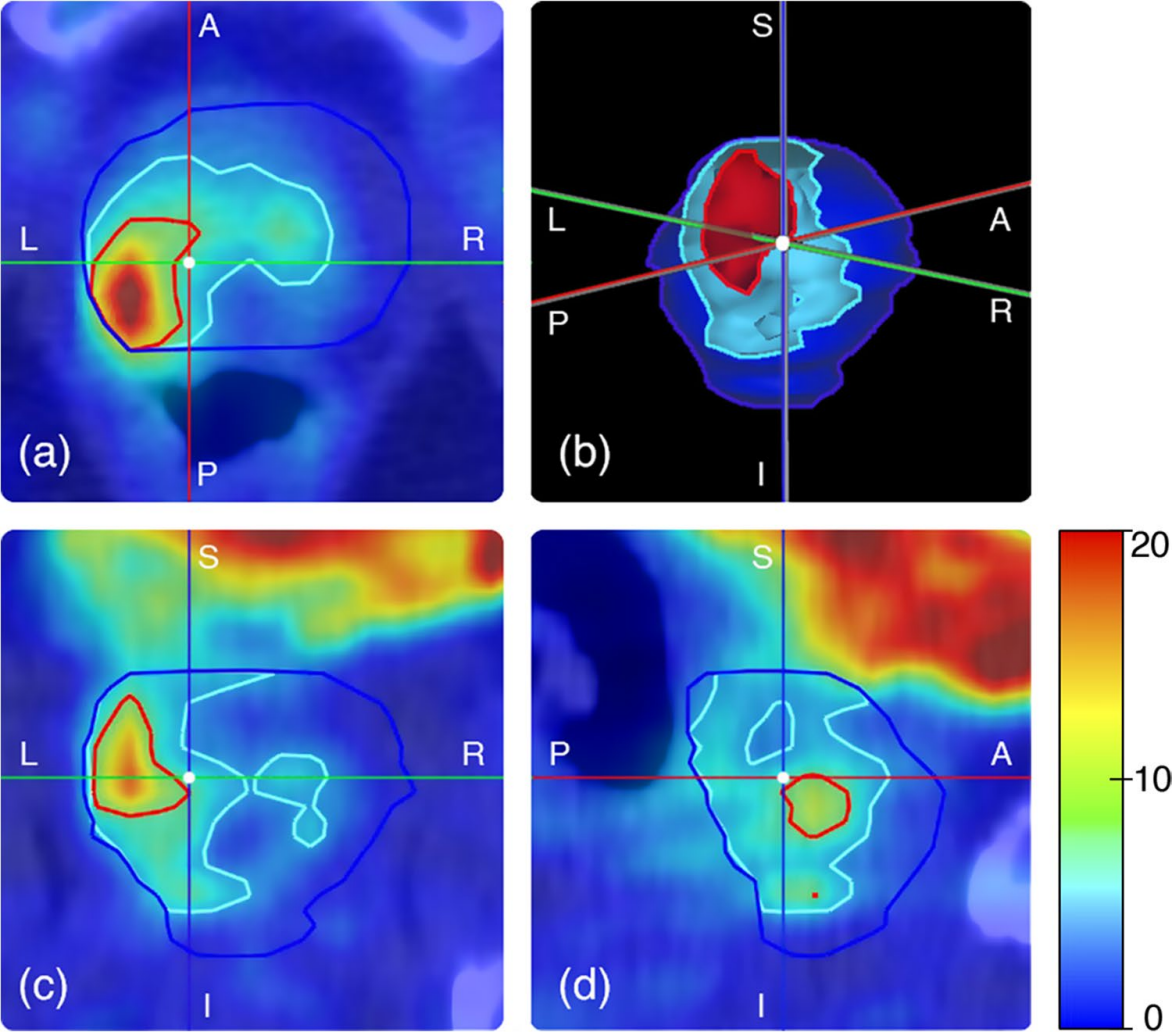
Fused PET and CT images of a representative patient in axial (**a**), coronal (**c**), and sagittal (**d**) views. High bladder uptake is visible in the upper portion of panels (**c**) and (**d**) as a non-homogeneous red–orange-yellow area. Panel (**b**) shows the corresponding 3D VOIs. In the figure, the blue line represents PG_whole_, the light blue represents PG_2.5_, and the red one PG_41%_ (S = superior, I = inferior, P = posterior, A = anterior, R = right, L = left)

The whole prostate gland (PG_whole_) was jointly delineated by two expert physicians (L.E., and P.G. with more than 10 and 4 years of experience in [^18^F]fluoromethylcholine PET/CT reporting, respectively) by manually segmenting, in a slice-by-slice fashion, the CT data of the hybrid imaging. Validation of the resulting segmentations was performed by cross-checking. Delineations were subsequently transferred to the PET data and, whenever necessary, they were refined to exclude from the segmentation the uptake due to the spill-out effect of the tracer accumulated in the bladder. Two additional segmentations were obtained by applying two conventional thresholds to the standardized uptake values (SUV) inside the PG: i.e., 41% of the maximum SUV value inside the prostate (PG_41%_), and SUV > 2.5 (PG_2.5_). The resulting regions (i.e., PG_whole_, PG_2.5_, PG_41%_) were considered separately for the analysis. Figure 1 shows the three segmentation approaches, for a representative patient.

### Radiomic features

Radiomic feature extraction was separately performed on the three different PG segmentations using the open-source and IBSI-compliant software S-IBEX [24, 25]. PET images were linearly interpolated to obtain an isotropic voxel size of 2 mm and re-segmented in [0–20] SUV range. To compute features requiring SUV discretization, the fixed bin size (FBS) method was chosen using bin widths of 0.2, 0.4, and 0.6 SUV, which resulted in 3 different feature sets for each PG segmentation method, for a total of 9 combinations. Further details regarding feature extraction are reported in the supplementary Table S1 and S2.

Each combination of PG delineations/bin widths included 172 radiomic features, belonging to 11 feature families [26], describing the shape, intensity distribution, and textural characteristics of the volume of interest (VOI). Finally, each patient had 9 different radiomic feature sets.

### Logistic regression models

**Fig. 2:**
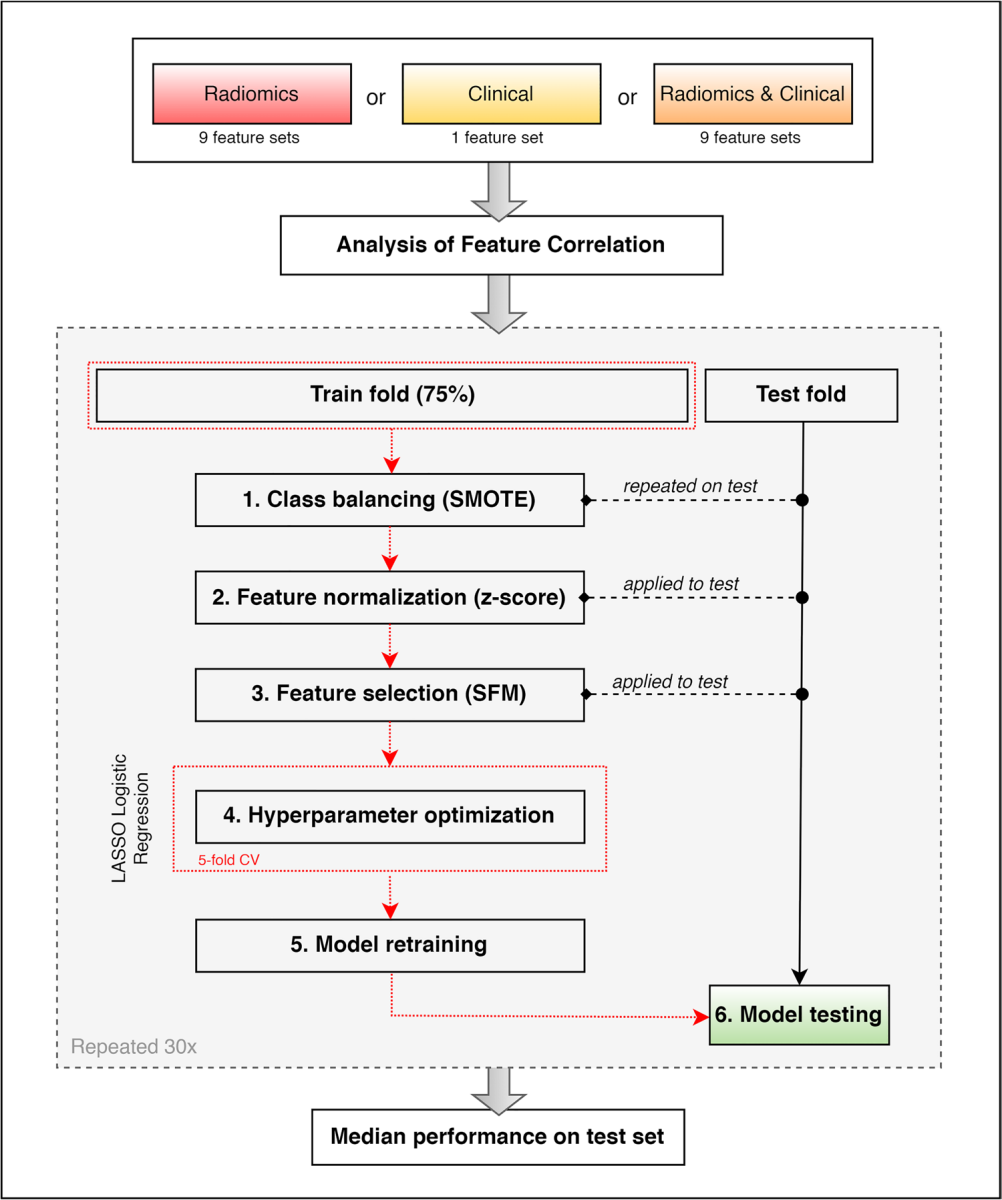
Training scheme for the LASSO logistic regression model

A unique standard pipeline was designed for the training of the prediction model and is depicted in Fig. 2. At first, we considered only clinical data to train the baseline model and assess whether the available clinical parameters alone enclosed predictive information for BCR. Subsequently, clinical data were integrated with each of the nine radiomic feature sets, given by PG delineation/bin width. Eventually, the performances of models trained with radiomic features alone have been also assessed for comparison.

At first, features with absolute Spearman’s correlation coefficient greater than 0.95 were removed from the dataset to reduce redundancy among predictors. The remaining features were fed to a logistic regression model to predict the BCR in a 30-repeated hold-out validation procedure, with a train-test ratio of 3:1. For each training phase, we repeated the following steps:

1. Data balancing with the synthetic minority oversampling technique (SMOTE) [27].
2. Feature normalization using Z-score.
3. Feature selection with “SelectFromModel” method of Scikit-learn Python library on the training set.
4. Training of a logistic regression model combined with the least absolute shrinkage and selection operator (LASSO) to further select the most informative parameters and predict BCR. A 5-fold cross-validation procedure was employed on the training set to optimize the regularization parameter lambda.
5. Model retraining on the whole training set using the optimal lambda.
6. Evaluation of prediction results through the area under the receiver operating characteristic (ROC) curve (AUC), as well as balanced accuracy, specificity, sensitivity, precision, and F1 score, where the F1 score is the harmonic mean of model precision and sensitivity.

For all metrics, median and 5th–95th percentiles on the 30 test sets of the validation procedure were derived. The entire analysis was implemented in Python programming using Scikit-learn and SciPy libraries (version 3.7).

## Results

For our cohort, the median PSA (that was missing for 2 patients) was 11 ng/mL (range 2.54–80.9 ng/mL), GS was ≤ 7 for 34 (46%) and GS > 7 for 40 (54%) patients, the clinical stage was T1 or T2 for 66 (89%), T3 for 7 (9%) patients, and missing for 1 (1%). Thirty-nine patients (53%) were treated with radical prostatectomy (with or without pelvic lymphadenectomy), while 35 (47%) underwent definitive radiotherapy. The BCR occurred in 28 (38%) patients. Median follow-up was 35.5 months (range 3.8–94 months). The PG_whole_ dataset included all 74 patients. Instead, 2 and 4 patients for PG_2.5_ and PG_41%_, respectively, were discarded because their VOIs presented a volume smaller than 0.5 cm^3^, which was not sufficient for a robust texture characterization of the volume of interest.

### Biochemical recurrence prediction

For each feature set, defined by segmentation approach and bin width, the median and [5th–95th] percentiles of the performance metrics of the LASSO logistic regression model are summarized in Table 1 for the baseline clinical model and for the models trained with both clinical and radiomic features and in Table S3 of the supplemental material for the ones based on radiomic features alone. The top five most selected features in the 30 folds of the developed pipeline are shown in Supplemental Fig. S1.

**Table 1:** Medians and [5th–95th] percentiles of the prediction results on the 30 test set folds for each segmentation/bin size feature set considering clinical or radiomic + clinical features (*AUC* area under the ROC curve; *FBS* fixed bin size; *PG* prostate gland)

		Clinical data only	PG_whole_			PG_2.5_			PG_41%_		
			FBS 0.2	FBS 0.4	FBS 0.6	FBS 0.2	FBS 0.4	FBS 0.6	FBS 0.2	FBS 0.4	FBS 0.6
AUC	Median	0.73	0.67	0.66	0.62	0.66	0.78	0.69	0.69	0.72	0.72
	5th- 95th percentiles	0.47–0.84	0.47–0.92	0.51–0.89	0.43–0.9	0.42- 0.91	0.62–0.88	0.35–0.9	0.47–0.88	0.41–0.89	0.47–0.91
Balanced accuracy	Median	0.69	0.65	0.65	0.65	0.67	0.75	0.67	0.69	0.69	0.71
	5th- 95th percentiles	0.5–0.81	0.5–0.88	0.56–0.83	0.56–0.87	0.54–0.86	0.62–0.86	0.5–0.86	0.56–0.83	0.54–0.83	0.56–0.85
Specificity	Median	0.69	0.77	0.77	0.69	0.83	0.83	0.92	0.88	0.92	0.92
	5th- 95th percentiles	0.38–1	0.31–1	0.46–1	0.34–1	0.54–1	0.5–1	0.54–1	0.46–1	0.46–1	0.45–1
Sensitivity	Median	0.69	0.69	0.65	0.69	0.58	0.75	0.58	0.65	0.62	0.58
	5th- 95th percentiles	0.11–0.92	0.27–0.85	0.15–0.85	0.22–0.92	0.16–0.83	0.37–0.83	0.08–0.8	0.19–0.85	0.08–0.89	0.15–0.85
Precision	Median	0.69	0.7	0.73	0.68	0.75	0.8	0.8	0.79	0.88	0.84
	5th- 95th percentiles	0.5–0.95	0.5–1	0.58–1	0.56–1	0.6–1	0.61–1	0.23–1	0.6–1	0.57–1	0.55–1
F1 score	Median	0.69	0.64	0.64	0.67	0.65	0.72	0.63	0.68	0.68	0.7
	5th- 95th percentiles	0.19–0.82	0.36–0.88	0.27–0.82	0.35–0.86	0.24–0.85	0.54–0.84	0.13–0.83	0.3–0.81	0.14–0.82	0.24–0.83

**Fig. 3:**
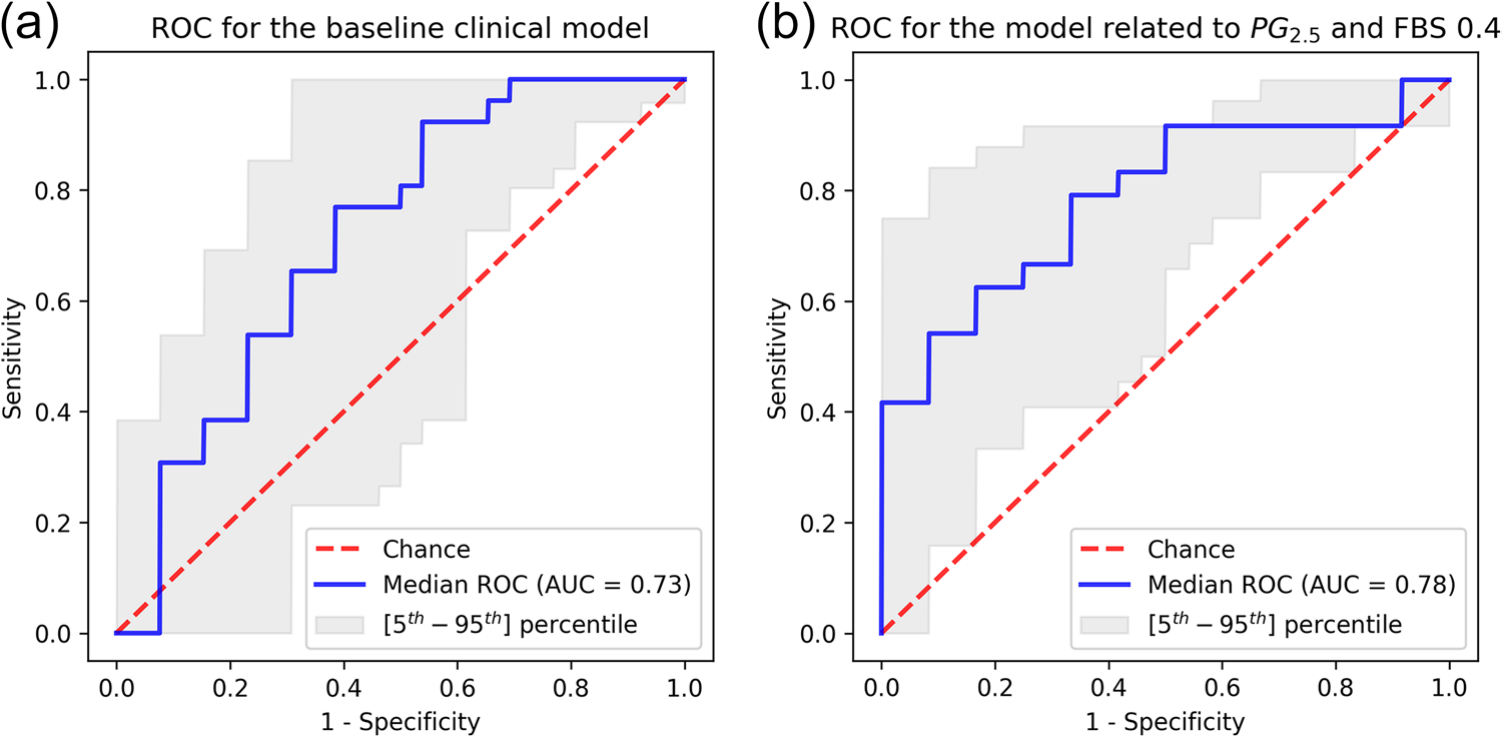
(**a**) Median ROC curve of the baseline clinical model and (**b**) for the model trained on PG_2.5_ 0.4 SUV considering radiomic and clinical features (ROC = Receiver Operating Characteristics curve; AUC = area under the ROC curve; FBS = Fixed Bin Size; PG = Prostate Gland)

The baseline clinical model achieved good performances with a median AUC of 0.73 (Fig. 3a). The highest performance scores were obtained by the model trained on PG_2.5_ volumes using a bin size of 0.4 SUV. For the model, the median AUC on the 30 test folds was 0.78 and median balanced accuracy, specificity, sensitivity, precision, and F1 score were 0.75, 0.83, 0.75, 0.80, and 0.72, respectively (Fig. 3b).

PG_whole_ had the lowest performances: for all bin sizes median AUC was lower than 0.70 and all other metrics obtained similar scores. PG_41%_ segmentation reached AUC values of 0.72 for 0.4 and 0.6 bin widths. For this approach, all bin widths obtained good scores in terms of specificity and precision, but at a price of reduced sensitivity. Overall, considering all ten feature sets, sensitivity was the metric with the lowest median and percentile scores. The highest value for sensitivity, equal to 0.75, was obtained for the model related to PG_2.5_ and FBS 0.4 SUV, followed by the baseline clinical model, where sensitivity was equal to 0.69. On the contrary, specificity achieved the highest median scores (up to 0.92) for almost every feature set.

Compared to the other models (except for PG_2.5_ and FBS 0.4 SUV), clinical data alone obtained good prediction results, with improved performance for the median AUC of 0.73, and the median F1 score of 0.69, but with worse results for the specificity.

Models trained with radiomic features only confirmed that the best combination was the one formed by PG_2.5_ segmentation and FBS 0.4 SUV for almost all metrics and that radiomic features alone contain predictive information for the BCR (Supplemental Table S3). Nevertheless, the importance of including clinical features in the model is supported by the fact that, for all models, GS and PSA were the most frequently selected features, being included in the model more than 20 out of 30 times in the model validation procedure (Supplemental Fig. S1). Furthermore, the resulting best model was able to point out the radiomic features that mostly contributed to the BCR prediction. Besides the GS selected 30/30 times, the model identified the “center of mass shift” of the morphological feature family, and the “maximum histogram gradient intensity” of the intensity histogram feature family, as equally important predictors of BCR, both included in the model 30/30 and 28/30 times.

## Discussion

In the present study, we tested the utility of radiomic analysis for the prediction of BCR in a cohort of intermediate and high-risk PCa patients undergoing [^18^F]fluoromethylcholine PET/CT at the initial staging of disease.

The results of our analysis show that, with respect to the baseline clinical model, based on PSA, GS, and clinical stage, BCR prediction performance further increases when clinical data are complemented with radiomic features. In particular, we found that the combination of a specific PG segmentation (PG_2.5_) with a 0.4 SUV discretization approach is the best way to process the original PET image in the view of the prediction of BCR. This means that discarding low SUV values inside the prostate by setting a 2.5 SUV threshold is beneficial for the analysis: more precise with respect to considering the whole PG and more conservative than the 41% threshold. Similarly, in the study by Tu et al. [28], the authors went beyond the traditional tumor-centric view of radiomic analysis and divided the whole prostate organ of 77 patients into three radiomic zones: the metabolic tumor zone, the proximal peripheral tumor zone, and the extended peripheral tumor zone inside the imaging boundaries of the organ. The authors found that these zones have different predicting strengths in classifying risk groups. Their study supports the hypothesis that radiomic features extracted from choline PET images can be predictive of several clinical endpoints and shows that, depending on the outcome, the useful information might be confined in specific areas of the gland.

As for the bin width used for SUV discretization, it resulted that the trade-off between the investigated bin sizes was the most successful. This may be due to the fact that using smaller steps may reduce the beneficial noise-suppressing property of discretization, while larger steps may determine an information loss, with different intensity values being condensed within the same bin, thus becoming indistinguishable.

To the best of our knowledge, our study is the first that correlates radiomic features to BCR events using [^18^F]fluoromethylcholine PET/CT. Indeed, some papers are now available about the use of radiomic models to predict the aggressiveness of PCa by using both radiolabeled choline and PSMA PET/CT or PET/MRI, while few data about the outcome are at disposal [20, 28–30].

However, growing evidence supports the use of risk stratification tools that combine clinical parameters, genomic biomarkers, and morphological and functional features able to either optimize health care or predict BCR in PCa patients [31–34]. Nevertheless, the lack of validation of these predictive tools in prospective randomized clinical trials represents the main limitation of their introduction in clinical practice. Methodology standardization, data sharing, and software accessibility are deemed additional important factors to increase the applicability and reuse of published studies. In this work, we adopted an open-source and highly standardized radiomic software, S-IBEX [25], to perform a complete and reproducible radiomic feature extraction.

### Limitations

This study has some limitations. First, it is built on a single-center cohort as other studies in the field [29, 35]. Nevertheless, the approaches we used for data preparation (i.e., redundancy reduction through correlation analysis, feature selection, class imbalance correction) as well as the cross-validation scheme implemented, minimized the chances of biased results, increasing generalizability and allowing to handle the relatively small sample size. The cross-validated prediction results indicate that our model was able to identify patients at risk of BCR in independent data.

Second, patients’ management included two diverse types of treatments (i.e., surgery or radiotherapy). However, for the purpose of this study, treatment did not affect the validity of prediction results since previous studies have demonstrated that, independently from the curative treatments, outcomes are analogous for patients with high-risk PCa [36, 37].

Additionally, [^18^F]fluoromethylcholine is a non-specific tracer for prostate cancer, and currently, PSMA-targeted radiotracers are considered more promising. However, they are also more expensive and have limited availability with respect to radiolabeled-choline (e.g., both ^18^F- or ^68^Ga-labeled PSMA have to be produced in loco, ^18^F-labeled PSMA is not commercially available yet, and ^68^Ga-labeled PSMA cannot be delivered far from the site of production due to the short half-life of ^68^Ga). As a consequence, PSMA-PET is currently performed in a relatively small number of diagnostic centers and we believe that [^18^F]fluoromethylcholine will remain the most widespread radiopharmaceutical for prostate cancer imaging for a long time to come. Nevertheless, the best-performing model thresholded PG uptake (i.e. SUV < 2.5), effectively discarding low SUV values that could be imputed to imaging limitations or to the non-specificity of [^18^F]fluoromethylcholine.

### Conclusion

In conclusion, this study demonstrates the feasibility of radiomic analysis of PET imaging to extrapolate useful information for the stratification of patients at risk of BCR.

In future studies, we aim to investigate the validity of proposed methods with novel tracers and imaging approaches such as PET/MRI using ^68^Ga-labeled PSMA ligands. However, prospective, multicentric studies are needed to investigate the clinical application of our findings and to fully explore the role of PET radiomics in clinical practice. Integration of clinical data, biochemical parameters, and radiomic features may greatly act as a multi-modal system to add prognostic information at the initial staging of PCa with the final purpose of addressing a tailored treatment strategy.

### Supplementary Information

Below is the link to the electronic supplementary material.

Supplementary file1 (PDF 306 KB)

## Abbreviations

AI: Artificial intelligence
AUC: Area under the receiver operating characteristic curve
BCR: Biochemical recurrence
CT: Computed tomography
FBS: Fixed bin size
GS: Gleason score
LASSO: Least absolute shrinkage and selection operator
MRI: Magnetic resonance imaging
PCa: Prostate cancer
PET: Positron emission tomography
PG: Prostate gland
PSA: Prostate-specific antigen
PSMA: Prostate-specific membrane antigen
ROC: Receiver operating characteristic
RP: Radical prostatectomy
RT: Radiotherapy
SMOTE: Synthetic minority oversampling technique
SUV: Standardized uptake value
VOI: Volume of interest

## Declarations

### Guarantor

The scientific guarantor of this publication is Dr. Laura Evangelista.

### Statistics and biometry

One of the authors has significant statistical expertise.

### Study subjects or cohorts overlap

Some study subjects have been previously reported in:

Sepulcri, M.; Fusella, M.; Cuppari, L.; Zorz, A.; Paiusco, M.; Evangelista, L. Value of 18F-fluorocholine PET/CT in predicting response to radical radiotherapy in patients with localized prostate cancer. *Clin. Transl. Radiat. Oncol.* 2021, *30*, 71–77, doi:10.1016/j.ctro.2021.07.002.

### Methodology

• Prospective

• Observational

• Performed at one institution
