# Supplementary material for: Role of radiomic analysis of [18F]fluoromethylcholine PET/CT in predicting biochemical recurrence in a cohort of intermediate and high risk prostate cancer patients at initial staging
Source: Eur Radiol. 2023 Apr 20;33(10):7199–208. doi: 10.1007/s00330-023-09642-9 (PMC10511374; doi:10.1007/s00330-023-09642-9)
Supplement: Supplementary file 1 — Supplementary file1 (PDF 306 KB) [file 330_2023_9642_MOESM1_ESM.pdf]

SUPPLEMENTAL MATERIAL

**Pre-processing step**	
Image Interpolation	
• *Algorithm*	Trilinear
• *Interpolation grid positioning*	align grid centers
• *Dimensions of the interpolation grid*	as per IBSI manual
• *Voxel spacing [mm]*	2 x 2 x 2
• *Intensity rounding*	rounded to nearest integer
ROI Interpolation	
• *Algorithm*	Trilinear (default)
• *Threshold*	0.5 (default)
Re-segmentation	
• *Range [SUV]*	[0 20]
Discretization	
• *Method/parameter (texture and IH)*	FBS: 0.2, 0.4 or 0.6 SUV
• *Method/parameter (IVH)*	FBS: 0.02, 0.04 or 0.06 SUV

Table S1. Parameters for the image pre-processing steps.

Parameter name	***Parameter value***
**GLCM**	
• *Aggregation methods*	3D:avg
• *Direction vectors*	13 direction for 3D aggregation
• *Symmetry*	yes
• *Distance*	1 (Chebyshev)
• *Distance weighting*	1 (default)
**GLRLM**	
• *Aggregation methods*	3D:avg
• *Direction vectors*	13 direction for 3D aggregation
• *Distance weighting*	1 (default)
**GLSZM**	
• *Aggregation methods*	3D
• *Linkage distance*	1 (Chebyshev)
**GLDZM**	
• *Aggregation methods*	3D
• *Linkage distance*	1 (Chebyshev)
• *Zone distance norm*	default (Manhattan)
**NGTDM**	
• *Aggregation methods*	3D
• *Distance*	1 (Chebyshev)
• *Distance weighting*	1 (default)
**NGLDM**	
• *Aggregation methods*	3D
• *Coarseness*	0
• *Distance*	1 (Chebyshev)
• *Distance weighting*	1 (default)

Table S2. Parameters for the extraction of texture features.

		PG_{whole}			$PG_{2.5}$			$PG_{41\%}$		
		**FBS 0.2**	**FBS 0.4**	**FBS 0.6**	**FBS 0.2**	**FBS 0.4**	**FBS 0.6**	**FBS 0.2**	**FBS 0.4**	**FBS 0.6**
**AUC**	*median*	0.64	0.56	0.59	0.62	0.73	0.66	0.63	0.59	0.67
	5th - 95th prctile	*0.39 - 0.86*	*0.42 - 0.9*	*0.4 - 0.85*	*0.41 - 0.87*	*0.56 - 0.93*	*0.4 - 0.83*	*0.39 - 0.83*	*0.34 - 0.84*	*0.43 - 0.89*
**ACC**	*median*	0.62	0.6	0.62	0.65	0.71	0.67	0.63	0.65	0.69
	5th - 95th prctile	*0.54 - 0.81*	*0.5 - 0.87*	*0.52 - 0.79*	*0.54 - 0.83*	*0.58 - 0.86*	*0.52 - 0.79*	*0.52 - 0.77*	*0.52 - 0.83*	*0.52 - 0.83*
**SPEC**	*median*	0.77	0.85	0.85	0.92	0.92	0.92	0.88	0.92	0.88
	5th - 95th prctile	*0.27 - 1*	*0.29 - 1*	*0.34 - 1*	*0.45 -1*	*0.67 - 1*	*0.5 - 1*	*0.27 - 1*	*0.52 - 1*	*0.38 - 1*
**RECALL**	*median*	0.65	0.46	0.42	0.5	0.58	0.5	0.54	0.54	0.54
	5th - 95th prctile	*0.15 - 0.85*	*0.08 - 0.85*	*0.11 - 0.85*	*0.08 - 0.88*	*0.2 - 0.8*	*0.2 - 0.75*	*0.07 - 0.92*	*0.08 - 0.81*	*0.15 - 0.92*
**PREC**	*median*	0.68	0.68	0.68	0.8	0.86	0.81	0.74	0.8	0.83
	5th - 95th prctile	*0.52 - 1*	*0.5 - 1*	*0.51 - 1*	*0.57 - 1*	*0.64 - 1*	*0.53 - 1*	*0.25 - 1*	*0.51 - 1*	*0.52 - 1*
**F1 score**	*median*	0.64	0.54	0.54	0.6	0.67	0.59	0.63	0.62	0.64
	5th - 95th prctile	*0.24 - 0.8*	*0.14 - 0.85*	*0.19 - 0.78*	*0.15 - 0.81*	*0.33 - 0.85*	*0.33 - 0.77*	*0.11 - 0.77*	*0.14 - 0.8*	*0.23 - 0.81*

Table S3: Medians [5th - 95th percentile] of the prediction results on the 30 test set folds for each PG delineation/bin width feature set considering radiomic features alone.

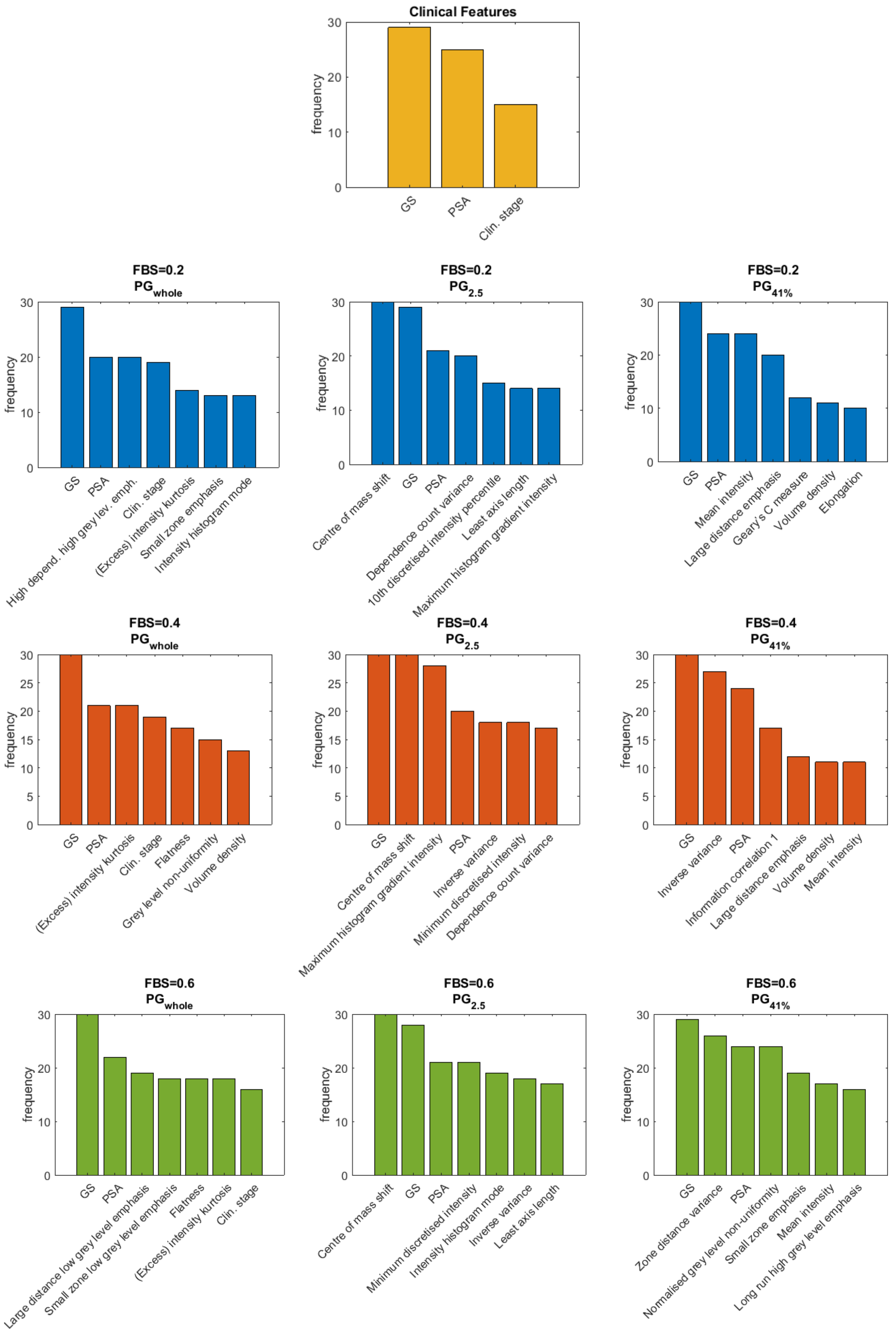

Figure S1. Top five most selected features by the LASSO logistic regression model, for the baseline clinical model and for each prostate delineation/bin width feature set.

Eur Radiol (2023) Marturano F, Guglielmo P, Bettinelli A et al
